# How the interplay between power concentration, competition, and propagation affects the resource efficiency of distributed ledgers

**DOI:** 10.1093/pnasnexus/pgag135

**Published:** 2026-05-26

**Authors:** Paolo Barucca, Carlo Campajola, Jiahua Xu

**Affiliations:** Centre for Blockchain Technologies, Department of Computer Science, University College London, Gower Street, London WC1E 6BT, United Kingdom; Institute of Finance and Technology, University College London, Gower Street, London WC1E 6BT, United Kingdom; Centre for Blockchain Technologies, Department of Computer Science, University College London, Gower Street, London WC1E 6BT, United Kingdom; Exponential Science Foundation, Lugano 6900, Switzerland

**Keywords:** carbon emission, sustainable distributed ledger technology, Bitcoin

## Abstract

Forks in the Bitcoin network arise as a natural consequence of competition within its Proof-of-Work consensus protocol, but lead to resource inefficiencies and compromise network security. The frequency of forks, therefore, serves as a critical indicator of a distributed ledger’s operational efficiency. We model the fork rate in a network of heterogeneous miners as a function of the number of miners, their hash rate distribution, and block propagation times within the peer-to-peer infrastructure. Empirical evidence demonstrates that fork rates are well-approximated by the ratio of the median block propagation time to mining time. Our model provides a theoretical foundation for this relationship while also capturing the fork rate’s additional dependency on miner heterogeneity. Our work establishes a robust mathematical setting for investigating factors often unobservable from existing empirical data, such as power concentration, competition, and asymmetric propagation times in distributed networks. Using this as a null model, we can detect anomalies in the historical fork rate—e.g. around 2016—indicating either high concentration of mining power or strongly heterogeneous latency in the Bitcoin network. We also estimate the mining power wasted in mining blocks on top of a nonlatest block, which potentiates accidental forks. The wastage amounts to approximately 16,000 MW in the most recent year, equivalent to half of the power generated in the United Kingdom.

Significance Statement Energy consumption in Proof-of-Work blockchains, such as Bitcoin, is a major concern for sustainability, attracting scrutiny from environmental scientists, policy-makers, and technologists. Although the ecological footprint of distributed ledgers is widely debated, rigorously quantifying inefficiencies due to fork events remains challenging. In this work, we introduce a mathematical framework modeling fork rates as functions of miner heterogeneity, network size, and block propagation delays. By mapping discarded blocks to energy consumption using empirical data, we reveal how network parameters drive wasted computing power. Our results offer actionable insights for protocol design and infrastructure optimization, supporting the development of more sustainable distributed ledger technologies.

## Introduction

The Bitcoin protocol (1), introduced in 2008, is the first and most famous blockchain-based system operating at scale. It consists of an append-only, public and permission-less ledger that records transactions initiated by pseudonymous addresses. While the content of the ledger is publicly available, write access to the ledger is secured by a cryptographic puzzle. This requires finding a string, called *nonce*, that is prepended to the new contents and passed through the SHA-256 function (2) to yield a hash below a given target value.

This puzzle can only be solved through brute-force calculation, and the winner of this race gains the right to append a new set of valid transactions (a block) to the most recent block and to create a predetermined amount of new Bitcoins that they can take as a reward. Engaging in this process is known as Proof-of-Work (PoW) mining, and its operators are called miners. The puzzle’s difficulty, namely how small the target value is below which the block hash must be, is periodically and automatically adjusted by the protocol so that on average a solution is found every 10 min, which is the target block time of the Bitcoin ledger. Given this mechanism, write access is also permission-less in principle: there is no central authority that has the right to censor or deny write access, and overwriting is also prohibited thanks to the protocol’s reliance on Merkle trees and chained hashes.

However, due to risk management and the strong economies of scale in technology markets (3), the competition for write access favors players who have enough economic resources to acquire large numbers of sophisticated purpose-built machines, known as Application-Specific Integrated Circuits or ASICs, which severely outperform commercial CPUs and GPUs in the solution of the PoW puzzle. This economic mechanism naturally pushes retail miners out of the mining market, as they end up having a negligible probability of successfully mining a block with respect to the most equipped and capitalized entities. Large mining operators also aggregate into even larger mining pools as a way to smooth out their reward payout and share the risk associated with the randomness of the PoW protocol.

This emerging concentration leads to a strongly heterogeneous distribution of hash rates, i.e. the hashes per second (H/s) that miners are able to generate.

At the time of writing, three mining pools produce over 50% of new Bitcoin blocks, which is a proxy for their share of the total hash rate committed to Bitcoin mining.^a^ This is concerning for several reasons: first, if a single entity could control the absolute majority of mining power, it would be able to run a 51% attack (4), i.e. to write false information on the blockchain while always producing the longest chain, which is the standard rule by which nodes determine the “validated” blockchain; second, as shown by (5), concentration distorts the fees market, which Bitcoin users have to pay to incentivize the miners to include their transaction in the limited space of the next block; third, it puts major miners in a censorship position, as they could arbitrarily delay the inclusion of specific transactions on the blockchain.

**Figure 1:**
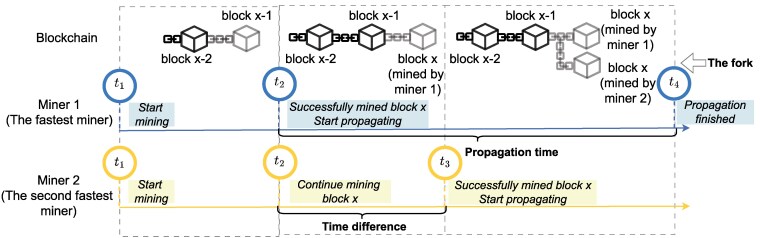
To simplify the dynamics, we assume that all miners start mining at $t_{1}$. At $t_{2}$, Miner 1 solves the PoW puzzle for block *x* and starts broadcasting it to the network. By $t_{4}$, the message has spread to most of the nodes and a new block x+1 is likely to be appended to block *x*. However, Miner 2 successfully mines the block at $t_{3}$, which is later than $t_{2}$ and earlier than $t_{4}$. Therefore, based on the definition above, the propagation time is $t_{4}-t_{2}$, and the fork is present between time $t_{3}$ and $t_{4}$.

In this paper, we explore the impact of the heterogeneity of the hash rate distribution on the probability of generating accidental forks. These situations, summarized in Fig. 1, arise when two miners solve the PoW puzzle almost simultaneously, and then have to compete to be the first to broadcast their block to the majority of other network nodes. This competition is due to the network latency, which in Bitcoin leads to an average propagation time of about 2 s to reach 90% of the network (Methods and data collection). An accidental fork (6, 7) can be resolved by the competition of the two versions of the block. The block which eventually is not included in the longest chain becomes an *orphaned* block, and its content will not be considered validated. Note that in Fig. 1 and for the sake of model tractability, we oversimplify the resolution of the fork at $t_{4}$, i.e. once Miner 1’s block has propagated to most of the network: in reality, these forks can take several downstream blocks to resolve by the longest chain rule. This simplification however does not significantly affect our model validity, as we focus on the occurrence of accidental forks rather than how long they can last, and linking fork resolution to network propagation times allows us to take advantage of statistics on network delays to apply our model to real data. Despite their indication of a distributed network, accidental forks are an inefficiency of the Bitcoin protocol that leads to wasted computational resources (and thus energy), increasing the cost of network operations and its environmental impact to maintain a given level of security.

We base our modeling approach on previous work by Fadda et al. (8), who investigate the impact of network structure on the generation of accidental forks. Our intent is to relax their assumption that all miners are equal, i.e. have the same hash rate, accounting for a more realistic hash rate distribution, while keeping our solution fully analytical to avoid cumbersome simulations. We use empirical data as well as literature estimates for the frequency of accidental forks in the Bitcoin blockchain (6), and compare it with the results of our modeling approach, which takes as input the number of miners and the parameters of the hash rate distribution.

Decker et al. (6) first investigated the fork generation from a network propagation perspective verifying that the primary cause of forks is indeed block propagation delay. The authors quantified the probability of the block mining time and fork generation with the assumption that the computational power is uniformly distributed. Remarkably, according to their simple model, the probability of forks was 1.78%, while their empirical data suggested a probability of 1.69%. Neudecker et al. (9) monitored empirical data and observed that the probability of a block being incorporated into the main chain has a roughly linear relationship with the time that block has been disseminated prior to the competing block. Shahsavari et al. (10) extended the fork probability equation further by introducing new factors, such as block size and network conditions, which determine the propagation time. They assumed the block arrivals as a homogeneous Poisson process and found that smaller block sizes and higher bandwidth have the effect of decreasing the fork rate, finding good agreement with empirical fork rates. Misic et al. (11) analyzed how network latency and geographic distribution affect fork frequency and blockchain growth dynamics in Bitcoin-like networks using simulations. Tessone et al. (12) proposed a stochastic model and performed numerical simulations to investigate the dynamics of blockchain-based consensus. They found that the concentration of mining power, measured by its Gini index, tends to decrease as network latency increases. Numerical simulations conducted by Liu et al. (13) showed that the fork rate increases with the number of miners, *N*.

As for the theoretical approach which is at the basis of our present contribution, Fadda et al. (8) estimated the probability of forks by computing analytically the probability that the time difference of the two fastest miners is lower than the propagation delay needed to reach the whole network. Based on the assumption of homogeneous miners, they find that their model tends to overestimate the frequency of accidental forks. This assumption is relaxed in the current paper, based on clear evidence from data and a number of studies, including (14), that presents a latency-aware efficiency model showing that communication delays can cause miner inequality in PoW networks. Further, in Cao et al. (15), the authors empirically measured how network latency advantages increase revenue and centralization risk among Bitcoin mining pools. From another perspective, the position of a node in the network can directly affect the efficiency of its mining, as studied in (16). Interestingly though, increasing connectivity is not always the optimal strategy to improve a node’s mining efficiency as shown in (17), as a node’s connections can aid also competing neighboring nodes.

Recent works have investigated how network asynchrony, topology, and miner incentives affect Bitcoin’s security, primarily from an adversarial perspective (18). Saad et al. showed that miners experience highly nonuniform block propagation delays; they exploit this to construct the HashSplit attack, whereby an adversary can orchestrate concurrent mining on multiple branches and violate common-prefix and chain-quality guarantees by selectively broadcasting blocks. In (19), the model incorporates realistic overlay topology and network churn into the security model and demonstrates SyncAttack, a double-spending strategy that does not require any mining power but instead exploits deteriorating synchronization and partitioning between reachable and newly arriving nodes. In (20), the authors provide a causal analysis of block propagation delays, identifying the growth of unreachable nodes, inefficiencies in the addressing protocol, round-robin block relaying, and high churn as key contributors to delayed dissemination, and propose protocol-level mitigations to improve synchronization. Finally, in (21), they analyze partitioning attacks that can isolate subsets of miners or delay consensus, thereby amplifying the feasibility of routing-based and topology-aware attacks. Studies on forks are closely related to the extensive literature on strategic mining behavior, like selfish mining (22), stubborn mining (23) or other practices (24). Eyal and Sirer (22) originally pointed out that the Bitcoin mining protocol is vulnerable to a potential attack where miners withhold their mined blocks, exploiting network delay and hash rate concentration to gain unfair advantages in mining sequences of blocks, rather than single blocks. Sarenche et al. (25) shift attention from network-layer attacks to incentive-driven mining strategies, analyzing how selfish mining, bribery, and mining-power distraction attacks can profitably destroy effective hash power and manipulate the difficulty adjustment mechanism in semi-rational environments with petty-compliant mining pools. Xiao et al. (26) proposed an analytical framework to evaluate the impact of network connectivity on PoW consensus security under adversarial scenarios. While detection of the practice has been scarce, recent research (27) has devised a statistical test that finds anomalous activity by miners that would be compatible with strategic mining. However, recently a slightly different and statistically undetectable type of selfish mining attack has been demonstrated to be viable on the Bitcoin blockchain (28). Other blockchains are not immune to similar attacks, for instance the timestamping attacks that have been detected on the Ethereum 1.0 blockchain (29). In contrast to these security- and incentive-driven studies, which frame forks and delays primarily as vectors for adversarial exploitation or profit-maximizing deviations, we model fork formation as the endogenous result of miner heterogeneity, hash-rate concentration, and propagation delays in the absence of explicit attacks. Their contribution is a quantitative theory of “natural fork rates” and resource inefficiency, linking market concentration and latency to wasted computational effort through analytically tractable expressions calibrated on empirical data, rather than characterizing worst-case adversarial strategies or protocol vulnerabilities.

In the following, we show the emergence of mining pools in the past decade and are able to interpret their macroscopic effects on the evolution of propagation time and fork rates. We demonstrate that while the ratio between the block propagation time and the mining time is a sufficiently accurate estimator of the fork rate, nonlinear effects arise due to the heterogeneity of miner activity that require more sophisticated estimators. Further, we show the emergence of a truncated power law (TPL) distribution in hash rates and its consequences for the fork rate. Finally, we quantify power wasted in producing blocks mined on top of a nonlatest block. Overall, we provide a quantitative model for investigating and interpreting the consensus dynamics on a distributed network and for designing future and alternative scenarios for existing and new blockchain mining systems.

## Results

**Figure 2:**
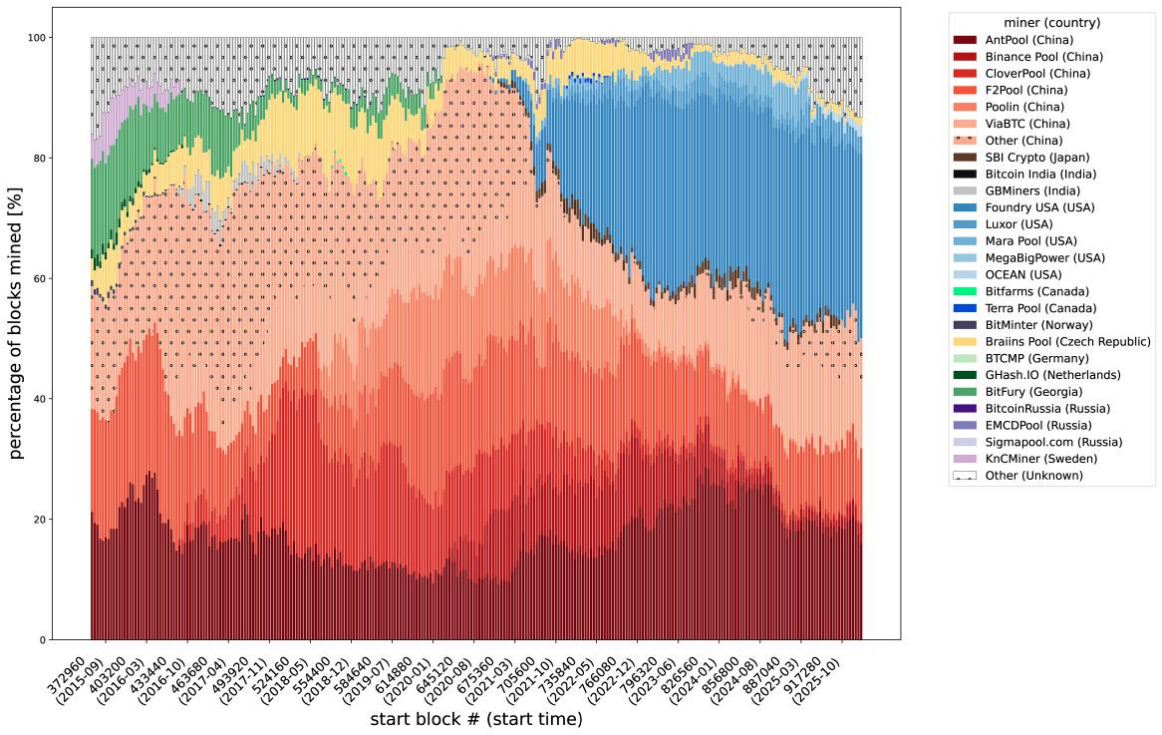
Share of blocks mined among miners in selected observation periods. Unknown miners, for which no specific signature is available, are in dotted white (top of figure), and the other minority China miners are in dotted light red.

Our first result is the estimation of the empirical distribution of hash rates and its evolution through the last decade. Dominance of few pools in the Bitcoin mining ecosystem is a well-known fact, and we qualitatively confirm it with our descriptive analysis. Figure 2 shows the dominance of mining pools based in China, such as Antpool and F2Pool. Since 2022, some of those pools phased out—likely due to the China’s Bitcoin Mining Ban in 2021—and US pools such as Foundry USA started to take the lead.^b^ We report summary statistics of selected observation periods in Table S1a, and data covering the entire sample in Supplementary data, Empirical and fitted distributions of hash rates.xlsx.

Figure S5 exhibits the distribution of hash rates $\lambda_{i}$ of several time periods. We compare the empirical probability to a set of fitted null distributions, namely the exponential $\lambda_{i}∼\mathrm{Exp}(r)$, the log-normal $\lambda_{i}∼\mathrm{LN}(\mu ,\sigma^{2})$ or the TPL $\lambda_{i}∼\mathrm{TPL}(\alpha ,\beta )$, as well as a semi-empirical Bayesian posterior that accounts for the potential difference between the estimator we use (i.e. the share of mined blocks) and the actual share of hashing power. The displayed distributions all consider i.i.d. hash rates; the null distributions are fitted using the method of moments Eq. 15, while the semi-empirical one was computed according to Eq. 6. We further motivate our choice of null distributions and the estimation techniques for their parameters in the Methods and data collection section.

Among the null distributions, the log-normal and TPL are the closest fit, both able to capture the right tail—with the TPL distribution slightly more so—better than exponential. However, the semi-empirical distribution is better at capturing the tail thickness and cut-off. These facts are well-known from a growing body of literature on the emerging inequality in cryptocurrencies (14, 15); however, the direct effect of the heterogeneity in mining power on the mathematical modeling of the probability of fork rates has not been studied so far. The main goal of our work is to develop such a model, and quantify the impact of hashrate and geographical concentration on the emergence of accidental forks. Having such a model not only allows to understand the consequences of centralization but also to calculate the implied primitives of the model based on the available data on forks and propagation times, which can then be used for anomaly detection. In the following paragraphs, we introduce a minimalistic model and describe how it can be used to connect fork rates, network latency and inequality in hashrates.

### Modeling consensus propagation in a heterogeneous network

We assume that the number of successful mining events occurring in a fixed time period is Poisson distributed, as the continuous time limit of a Bernoulli process with low enough success probability. Therefore, in our model, the time to successfully mine a block is exponentially distributed, and the probability of a block being mined at time *t* (assuming $t_{1}=0$) for miner *i* is


(1) $$p_{i}(t)=\frac{\lambda_{i}}{e^{\lambda_{i}t}},$$

where $\lambda_{i}$ represents the hash rate of miner *i*, i.e. the expected number of blocks per unit of time the miner can independently mine (i.e. without the influence of competition). The minimum time for any miner to succeed mining a block, $t_{\min}$, is the first order statistic $t_{\left(1\right)}$ of the system of miners, which has probability distribution function


(2) $$p_{\left(1\right)}(t|\{\lambda_{i}\})=\sum_{i=1}^{N}\left(\;p_{i}(t)\prod_{\;j\neq i}^{N}S_{j}(t)\right),$$

where $\left\{\lambda_{i}\}=\{\lambda_{1},\ldots ,\lambda_{N}\right\}$, *N* is the number of miners, and $S_{j}(t)=\int_{t}^{\infty }p_{j}(s)ds=e^{-\lambda_{j}t}$ is the probability that miner *j*’s mining time exceeds *t*. Plugging exponential distributions in the equation, it reads


(3) $$p_{\left(1\right)}(t|\{\lambda_{i}\})=\Lambda e^{-\Lambda \cdot t},$$

where $\Lambda =\sum_{i}\lambda_{i}$ represents the aggregate mining power of the network. It is then clear that the expected minimum mining time in this setup reads


(4) $$\left\langle t_{\min}\right\rangle =\frac{1}{\Lambda }$$

that, for the Bitcoin network, is kept at 10 min by the adjustment of the PoW puzzle difficulty parameter. To estimate the probability of an accidental fork to occur, we need to obtain the distribution for the time difference between the two fastest miners, which can be obtained from the joint distribution of the first and second order statistics:


$$\begin{array}{rl} \;p\left(t,t^{\prime }|\{\lambda_{i}\}\right) & =\sum_{i\neq j}\theta (t^{\prime }-t)p_{i}(t)p_{j}(t^{\prime })\prod_{k\neq i,j}^{N}S_{k}(t^{\prime })\\ & =\sum_{i\neq j}\frac{\theta (t^{\prime }-t)\lambda_{i}\lambda_{j}}{e^{\lambda_{i}(t-t^{\prime })+\sum_{k}\lambda_{k}t^{\prime }}}, \end{array}$$

where $t^{\prime }$ is the second shortest mining time and *θ* is the Heaviside step function, equal to 1 for $t^{\prime }>t$ and 0 otherwise. The distribution of time differences $\Delta =t^{\prime }-t$ can then be derived as


(5) $$\begin{array}{rl} \;p(\Delta =t^{\prime }-t|\{\lambda_{i}\}) & =\int dt\;dt^{\prime }\;\delta (\Delta -t^{\prime }+t)\;p(t,t^{\prime }|\{\lambda_{i}\})\\ & =\frac{\sum_{i=1}^{N}\lambda_{i}\sum_{\;j\neq i}\lambda_{j}\;e^{-\Delta \sum_{\;j\neq i}\lambda_{j}}}{\sum_{i=1}^{N}\lambda_{i}}, \end{array}$$

where δ(x) is the Dirac delta function, which selects only those values of $t^{\prime }$ and *t* such that their difference equals *Δ*.

The probability of a time gap between the two fastest miners smaller than or equal to $\Delta_{0}$ can be computed as *Δ*’s cumulative probability,


(6) $$\begin{array}{rl} C(\Delta_{0}|\{\lambda_{i}\}) & =\int_{0}^{\Delta_{0}}p(\Delta |\{\lambda_{i}\})\;d\Delta \\ & =\frac{\sum_{i=1}^{N}\int_{0}^{\Delta_{0}}\frac{\lambda_{i}\sum_{\;j\neq i}\lambda_{j}}{e^{\Delta \sum_{\;j\neq i}\lambda_{j}}}\;d\Delta }{\sum_{i=1}^{N}\lambda_{i}}\\ & =\frac{\sum_{i=1}^{N}\lambda_{i}\left(1-\frac{1}{e^{\Delta_{0}\sum_{\;j\neq i}\lambda_{j}}}\right)}{\sum_{i=1}^{N}\lambda_{i}}\\ & =1-\frac{\sum_{i=1}^{N}\lambda_{i}/e^{\Delta_{0}\sum_{\;j\neq i}\lambda_{j}}}{\sum_{i=1}^{N}\lambda_{i}}. \end{array}$$

Since $C(\Delta_{0}|\{\lambda_{i}\})$ is the probability that an accidental fork occurs, $p(\Delta_{0}|\{\lambda_{i}\})$ represents the sensitivity of fork rate to propagation time $\frac{dC(\Delta_{0}|\{\lambda_{i}\})}{d\Delta_{0}}$. Specifically at Δ=0, this sensitivity can be simplified as


(7) $$\begin{array}{rl} \;p(0|\{\lambda_{i}\}) & =\frac{\sum_{i=1}^{N}\lambda_{i}\sum_{\;j\neq i}\lambda_{j}}{\sum_{\;j=1}^{N}\lambda_{j}}\\ & =\frac{\sum_{i=1}^{N}\lambda_{i}\left(\sum_{\;j=1}^{N}\lambda_{j}-\lambda_{i}\right)}{\sum_{\;j=1}^{N}\lambda_{j}}\\ & =\left(\sum_{i=1}^{N}\lambda_{i}\right)\left[1-\sum_{i=1}^{N}{\left(\frac{\lambda_{i}}{\sum_{\;j=1}^{N}\lambda_{j}}\right)}^{2}\right], \end{array}$$

where $\sum_{i}^{N}(\frac{\lambda_{i}}{\sum_{j}^{N}\lambda_{j}}{)}^{2}=:HHI\in (0,1]$ is the Herfindahl–Hirschman Index (HHI) of the mining network, describing the level of hash rate concentration: higher HHI represents a more concentrated distribution of hash rates among miners, whereas a lower value signifies a more equal distribution. At sufficiently small $\Delta_{0}$, $C(\Delta_{0}|\{\lambda_{i}\})$ can be estimated with its first-order Taylor series approximation,


(8) $$C(\Delta_{0}|\{\lambda_{i}\})\approx \Delta_{0}\cdot p(0|\{\lambda_{i}\})=\Delta_{0}\cdot \Lambda \cdot (1-HHI).$$

Eq. 6 represents conditional cumulative probability: we can then take an ergodic approach and consider the average over different realizations of hash rates, to obtain the unconditional one


(9) $$C(\Delta_{0})=1-\int_{D}p(\{\lambda_{i}\})\left(\frac{\sum_{i}^{N}\frac{\lambda_{i}}{e^{\Delta_{0}\sum_{\;j\neq i}\lambda_{j}}}}{\sum_{i}^{N}\lambda_{i}}\right)d\{\lambda_{i}\},$$

where $D\subset R_{+}^{N}$ is the *N*-dimensional hyperplane of $\left\{\lambda_{i}\right\}$’s viable range. When $\forall \;i\neq j,\lambda_{i}⊥\lambda_{j}$, we can write out $C(\Delta_{0})$ as


(10) $$\begin{array}{rl} C(\Delta_{0}) & =1-\int_{0}^{\infty }[\sum_{i=1}^{N}(\int_{0}^{\infty }\frac{\lambda_{i}\;p(\lambda_{i})}{e^{x\lambda_{i}}}\;d\lambda_{i}\\ & \;\times \prod_{\;j\neq i}\int_{0}^{\infty }\frac{\;p(\lambda_{j})}{e^{(\Delta_{0}+x)\lambda_{j}}}\;d\lambda_{j})]\;dx. \end{array}$$

The derivation of Eq. 10 can be found in SI Appendix, “Derivation of Eq. 10 in *Research Article*.” When we impose the additional assumption of identical distribution, i.e. $\left\{\lambda_{i}\right\}$ are i.i.d., we get


(11) $$\begin{array}{rl} C(\Delta_{0}) & =1-N\int_{0}^{\infty }[\left(\int_{0}^{\infty }\frac{\lambda \;p(\lambda )}{e^{x\lambda }}\;d\lambda \right)\\ & \;\times {\left(\int_{0}^{\infty }\frac{\;p(\lambda )}{e^{(\Delta_{0}+x)\lambda }}\;d\lambda \right)}^{N-1}]dx. \end{array}$$

### Implied versus empirical fork rates

**Figure 3:**
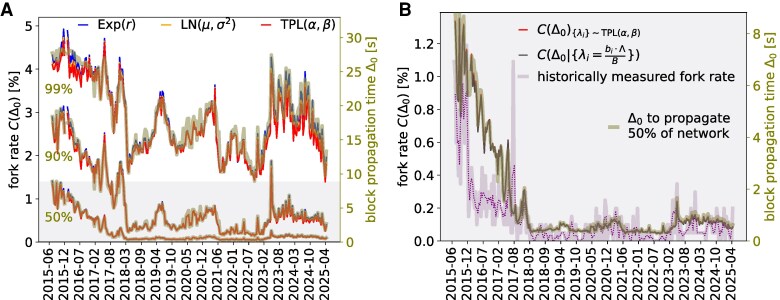
Time series of historically measured fork rates (dotted line) compared with model-estimated fork rates under the assumption of various distributions when different values of propagation time are used. The shaded area around the historical rates represents 90% confidence band. A) Model-estimated fork rates under different null distribution assumptions and at different levels of block propagation time $\Delta_{0}\in \{\Delta_{0}^{\left(50\right)},\Delta_{0}^{\left(90\right)},\Delta_{0}^{\left(99\right)}\}$. B) Model-estimated fork rates using TPL(α,β) versus using empirical $\left\{\lambda_{i}\right\}$, for $\Delta_{0}=\Delta_{0}^{\left(50\right)}$ network propagation time, compared with historically measured fork rates (dotted curve representing 3-period moving average).

The main advantage of having an analytical formulation of fork generation from mining and network data is that we can use it to validate its inputs based on the empirical frequency with which forks occur. In other words, we can identify under which combinations of $\Delta_{0}$ and $p(\{\lambda_{i}\})$ the fork rate is compatible with the empirically measured one, and compare them with the block propagation times and the share of mined blocks, respectively. In Fig. 3, we compare model-estimated fork rates using fitted parameters (Table S1b) with historical fork rates (SI Table S1a). To choose the threshold time $\Delta_{0}$, we use the estimates of the time it takes for a block to reach 50, 90, or 99% of the Bitcoin network (Fig. 3A), which we name $\Delta_{0}^{\left(50\right)}$, $\Delta_{0}^{\left(90\right)}$ and $\Delta_{0}^{\left(99\right)}$ respectively. We observe that exponentially distributed $\lambda_{i}$s yield the highest fork rate, followed by log-normally and then TPL-distributed hashrates, although the gap between the latter two is rather negligible.

We show in Fig. 3B that there is close to no difference between the estimated fork rate using a fitted distribution such as TPL(α,β) versus numerically solving the model with the empirically measured $\left\{\lambda_{i}=\frac{b_{i}\cdot \Lambda }{b}\right\}$, suggesting a high goodness of fit. Furthermore, the model-predicted fork rate is broadly consistent with the empirical estimates when $\Delta_{0}=\Delta_{0}^{\left(50\right)}$. Whilst the model more often overestimates than underestimates—particularly around 2016—it reproduces the temporal fluctuations (i.e., the “ups and downs”) observed in the data with good fidelity. Given that an exhaustive empirical record of stale and orphan blocks is unattainable, and that under-reporting is an inherent limitation of crowdsourced data, model-derived estimates are expected to exceed empirical measurements.

**Figure 4:**
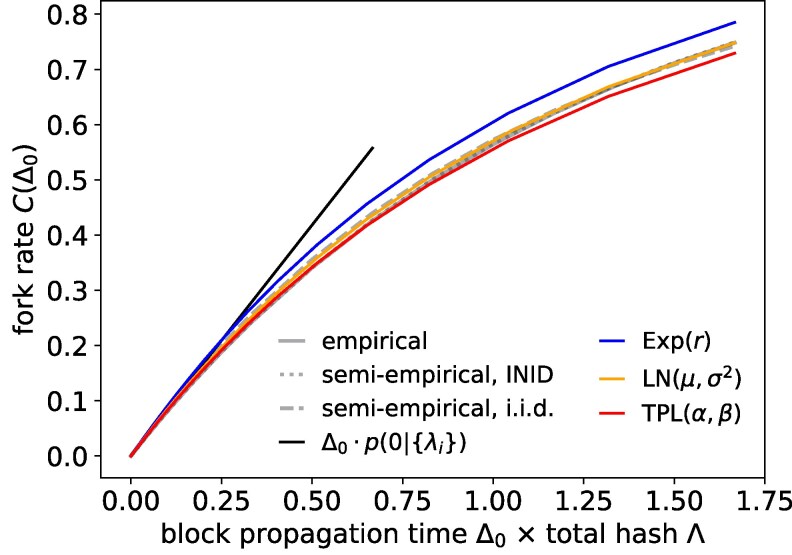
Fork rate estimation at various values of $\tau =\Delta_{0}\;\Lambda$. Comparison of fork rates obtained numerically via empirical CCDF, semi-empirical Bayesian estimates, and null distributions.

Figure 3B would suggest that the choice of null distribution has a marginal impact on the determination of the fork rate. To better investigate this, in Fig. 4, we compare the estimated fork rate under several distributional assumptions, applying Eqs. 10 and 11, and varying the characteristic time $\tau =\Delta_{0}\cdot \Lambda =\frac{\Delta_{0}}{\left\langle t_{\min}\right\rangle }$, i.e. the ratio between the block propagation time and the expected time to mine a new block. We can see that the difference between fork rates is barely distinguishable for τ<0.4, or $\Delta_{0}<240$ [s], in the case of Bitcoin, which is generally much larger than the real block propagation times, and that the linear approximation of Eq. 8 is largely valid over realistic ranges of *τ*. It is however good to notice that second-order effects start to become relevant at higher values of *τ*: these values are obviously rather unrealistic for Bitcoin, but may be attainable in faster PoW blockchains like Litecoin or Dogecoin, that operate with average block times of 2.5 and 1 min, respectively. At large $\Delta_{0}$, we see that the estimated fork rate is slightly higher when assuming $\lambda_{i}$s are independent but not identically distributed than under the i.i.d. assumption.

**Figure 5:**
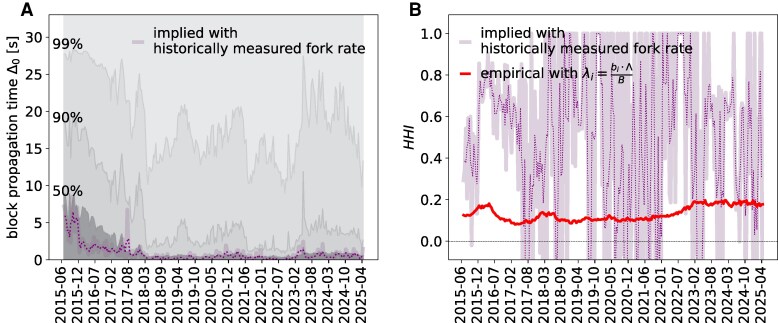
Time series of implied $\Delta_{0}$ and implied HHI versus their empirical values. A) implied $\Delta_{0}$ as $\frac{\text{historically measured fork rate}}{\Lambda \cdot (1-HHI)}$ compared with empirical block propagation time: to reach 50, 90, 99% of the network, respectively. B) implied HHI as $1-\frac{\text{historically measured fork rate}}{\Delta_{0}\cdot \Lambda }$ (dotted curve representing 3-period moving average) compared with empirical HHI using $\lambda_{i}=\frac{b_{i}\cdot \Lambda }{B}$.

These results suggest that the linear approximation of Eq. 8 is generally good enough to characterize the relation between $\Delta_{0}$, $p(\{\lambda_{i}\})$ and the fork rate. A very interesting result can be obtained by inverting this equation to calculate the “implied $\Delta_{0}$” and the “implied HHI,” i.e. the values of $\Delta_{0}$ and *HHI* that would produce the empirical fork rates. In Fig. 5A, we show the implied $\Delta_{0}$ given the historical fork rate and the measured HHI from the empirical $\left\{\lambda_{i}\right\}$, compared with the historical block propagation times $\Delta_{0}^{\left(50\right)}$, $\Delta_{0}^{\left(90\right)}$, and $\Delta_{0}^{\left(99\right)}$ of the network. We see how the implied $\Delta_{0}$ is very close to the median time it takes to broadcast a new block to Bitcoin nodes after mid-2017, while it is significantly smaller before that: this would suggest that miners have a connectivity within the network that is better than most nodes, i.e. they are among the first 50% of nodes that hear about a new mined block, thus avoiding the generation of forks. Moreover, the discrepancy prior to the summer of 2017 is likely at least in part explained by the “Compact Block Relay (Bitcoin Improvement Proposal 152)” update, which was being adopted by the network around that time and has likely reduced the latency advantage that some miner clusters may have had up to that point (30).

We also compute the HHI implied by the historically measured fork rate (Fig. 5B) assuming that $\Delta_{0}=\Delta_{0}^{\left(50\right)}$, and compare its time series with the HHI computed using empirically observed block mining data (SI Appendix, Eq. 2). We see that the implied HHI is significantly higher than the empirically measured HHI around 2016, 2019, and 2022; we also see how it sometimes drops to much lower and even negative values, which would challenge the interpretation of the indicator as an *HHI*. This is a consequence of fixing $\Delta_{0}=\Delta_{0}^{\left(50\right)}$: a negative implied *HHI* means that the $\Delta_{0}$ being used is too large.

**Figure 6:**
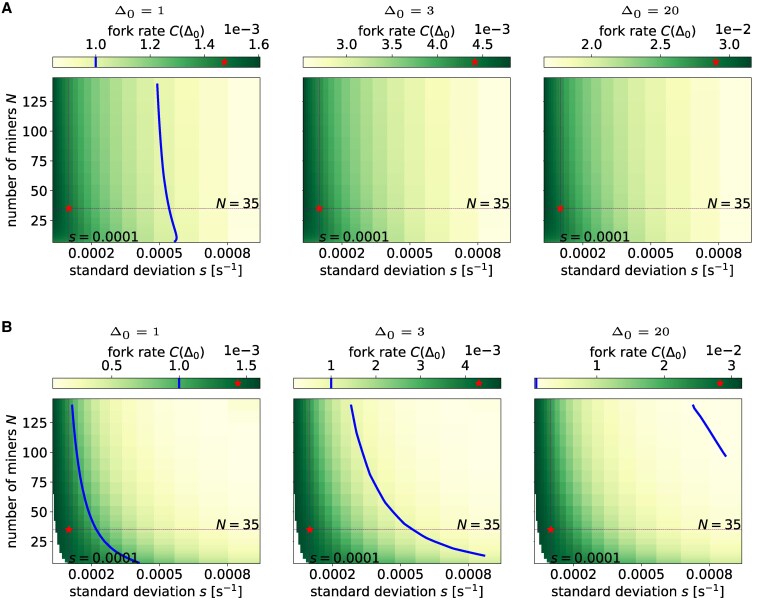
Extrapolation of fork rates under the log-normal or TPL hash rate distributions. Each subplot shows the fork rates with different miner numbers and hash rate standard deviation, at a given block propagation delay. The red star marks model-estimated fork rate at N=35, and s=0.0001; the blue isoline represents a fork rate of 0.1%. A) Log-normal. B) Truncated power law.

Given the above results, it now becomes clear that the discrepancy between model-implied and historically-measured fork rates seen in Fig. 3B can likely be explained by (a combination of) three factors: (i) a higher actual fork rate, (ii) a lower actual block propagation time ($\Delta_{0}$), and/or (iii) a higher power concentration, or lower competition (HHI), than the value that can be estimated by tracking block signing by miners. For the years before 2019, in particular, it may be because the most powerful miners around that period were geographically concentrated in one area, e.g. China (Fig. 2), and the communication between them was faster than between any two average nodes, hence lower actual $\Delta_{0}$ than the one we used to calculate fork rate. This would suggest that the Bitcoin peer-to-peer infrastructure might present core-periphery features, with a strongly connected core of nodes including the majority of miners, and a loosely connected periphery of nodes that do not contribute to the mining process. This centrality of miners has already been reported in economic analyses of transaction networks (31, 32), and these results would be consistent with a similar result for the peer-to-peer overlay. An alternative explanation would be that there has been sporadic collusion between miners, which would result in lower actual HHI. Finally, we examine how hash rates distribution heterogeneity, proxied by the standard deviation *s*, and the number of active miners *N* would affect fork rates. In Fig. 6, we show how fork rates change at different *s* under log-normal and truncated power law distribution of hash rates, varying the number of miners *N*, for a given total hash rate $\Lambda =0.0017s^{-1}$ across different block propagation times. We see that the fork rate diminishes as *s* increases, that is, when the hash power is more concentrated and heterogeneous. The number of miners *N* has a similar effect: the fork rate decreases as *N* increases, keeping *s* constant. This effect is more apparent under the TPL than under the log-normal distribution. The blue curve in Fig. 6 represents an isoline for a fork rate of 0.01%, with the red star reflecting situations observed toward the end of the sample period with Λ=0.0017, N=35 and $s=1\times 10^{-4}$.^c^ The analytically derived fork rate is close to the recently observed 0.1% at $\Delta_{0}=1$ but is above it when $\Delta_{0}$ is higher.

In summary, Fig. 6 shows the offsetting effects on fork rate from a decreasing number of miners *N* and increasing miner hash rate *s*. This explains why in Fig. 3 it appears that the block propagation time $\Delta_{0}$ is the only driver on model-estimated fork rates—it is because historically *N* and *s* have been moving in opposite directions ( Table S1a) and their respective effects on estimated fork rates have been canceling out.

### Effect of distributions for $\left\{\lambda_{i}\right\}$ on energy wastage

Assume a block’s fastest miner is miner *k*; then as soon as the block is mined, only miner *k* continues to mine on top of the mined block, whereas everyone else is mining on top of the previous block, while the mined block is being propagated. Therefore, we can calculate the “wasted” energy used to mine on top of the nonlatest block as $\sum_{i\neq k}\lambda_{i}\times \Delta_{0}$. Note that we consider all the power used to mine on top of the latest block to be “useful,” as it is needed by the design of the PoW consensus mechanism. Wastage only arises after a new block is mined, but other miners in the network continue to attempt the incorrect PoW puzzle unknowingly while the information on the newly mined block is still being transmitted.

It is thus apparent, and intuitive, that the energy wasted is positively proportional to the block propagation delay, $\Delta_{0}$. For each mined block, $\sum_{i\neq k}\lambda_{i}$ can thus be considered as “wasted hash,” whose expected value can be expressed as:


(12) $$E\left(\sum_{i\neq k}\lambda_{i}\right)=N\int_{0}^{\infty }dx\sum_{i\neq k}\int_{D}\lambda_{k}\lambda_{i}\prod_{\;j=1}^{N}\frac{1}{e^{x\lambda_{j}}}p(\{\lambda_{j}\})\;d\{\lambda_{j}\}.$$

When $\forall \;i\neq j,\lambda_{i}⊥\lambda_{j}$, we have


(13) $$E\left(\sum_{i\neq k}\lambda_{i}\right)=N\int_{0}^{\infty }dx(N-1)\left[{\left(\int_{0}^{\infty }\frac{\lambda p(\lambda )d\lambda }{e^{x\lambda }}\right)}^{2}{\left(\int_{0}^{\infty }\frac{\;p(\lambda )d\lambda }{e^{x\lambda }}\right)}^{N-2}\right].$$

The derivation of Eq. 12 and Eq. 13 can be found in SI Appendix, “Derivation of Eq. 12 and Eq. 13 in *Research Article*.”

**Figure 7:**
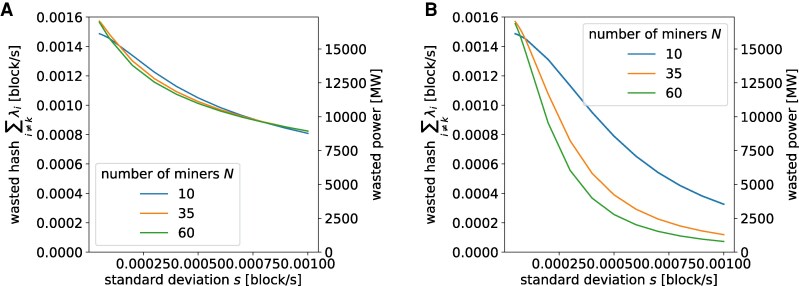
Effect of hash distribution type, standard deviation and number of miners on wasted hash per block $E(\sum_{i\neq k}\lambda_{i})$ at a given level of total hash rate *Λ* (Eq. 13). The corresponding wasted power (Eq. 14) is calculated using network difficulty and mining efficiency data as per 2025 January 1. A) Log-normal. B) Truncated power law.

In Fig. 7, we investigate, at a given total hash rate *Λ*, how various hash distributions affect wastage. To translate the results also into power, we source mining hardware efficiency values from the Cambridge Bitcoin Electricity Consumption Index^d^ and apply


(14) $$\begin{array}{rl} \text{wasted power [ W] } & =\underset{\mathrm{wasted}\;\mathrm{hash}\;[\mathrm{block}/s]}{\underbrace{E\left(\sum_{i\neq k}\lambda_{i}\right)}}\\ & \;\times \text{difficulty [ hash/block] }\\ & \;\times \text{efficiency [ J/hash] }. \end{array}$$

We observe that a higher level of heterogeneity (manifested as a greater standard deviation) can reduce wasted hashing power. Additionally, when all other things are equal, hash rate following a TPL distribution appears to result in lower wastage than that following log-normal distribution.

We further apply our computation Eqs. 13 and 14 to estimate historical power wasted, i.e. mining power that did not contribute to the building of the canonical Bitcoin ledger. As shown in Table S1c, the wasted hash rate—measured in wasted number of blocks per second [block/s], and estimated as i.i.d. under either the log-normal or TPL assumption—appears to remain relatively stable throughout history, and even slowly decrease since 2019 due to the increasing concentration of mining power. However, as the growth of the mining efficiency (measured in the reduction in the amount of Joule needed to generate a certain number of hashes [J/*T*Hash]) has been increasingly outpaced by the growth of mining difficulty (measured as the number of hashes needed to produce a block [*T*Hash/block]), the power wasted is also growing with the passage of time. In the most recent year, the estimated power wastage has reached about 16,000 MW, equivalent to half of the electric power generated in the United Kingdom (36,800 MW^e^).

## Discussion and conclusion

We designed a mathematical model that can effectively characterize the impact of hash rate heterogeneity and network delay on the occurrence of accidental forks in PoW blockchains. We considered a set of heterogeneous miners, and computed analytically the probability of observing a given time difference between the two smallest mining times by averaging over the distribution of hash rates between miners. The model takes as input the hash rate distribution, number of active miners and expected network delay in block propagation to calculate the probability that an accidental fork occurs. We explore several specifications of hash rate distributions, considering both ex-ante distributions fitted from empirical mining data and a nonparametric Bayesian empirical distribution. On one hand, the model fits nicely to the empirical observations and the predicted fork rates are comparable with the empirical stale block rate. On the other hand, where the two values differ, i.e. the fraction of forks is lower or higher than the modeled value, it gives an indication that some fundamental conditions of the protocol might be breached, e.g. miners not acting independently or miners having faster communication than most other nodes in the Bitcoin network. The model provides a fundamental relationship which allows the fast estimation and calibration of natural fork rates in blockchain systems, for arbitrary distributions of hash rates. In this framework, it will be possible to model more in detail the network propagation time, and as a consequence, the simultaneous modeling of the propagation process together with the mining process. This will allow not only to flag discrepancies between the real and theoretical fork rates that should emerge based on the distributions of hash rates in a set of independent miners, but also to associate these discrepancies to specific heterogeneous network properties of the miners. A practical application of the model is to quantify power wasted in mining blocks on top of a nonlatest block. We find this wastage to be drastically increasing over the past decade, peaking at around 16,000 MW in the most recent year, equivalent to half of the power generated in the United Kingdom.

Our study is not exempt from limitations. The most important ones are the lack of detail of intra-pool dynamics, the assumption that hash rates are constant within a difficulty epoch (2,016 blocks, or 2 weeks) and the relatively strong assumption that miners start mining at the same time. Another limitation lies with selecting a single value for $\Delta_{0}$, which implicitly assumes that blocks propagate at the same speed regardless of the mining node that mined them. Whilst these assumptions were necessary to get to our mathematical description, it is possible that at least some of them could be relaxed and we encourage further research on the topic.

Despite the limitations, we find a good agreement between the predicted fork rates, and in particular they are consistent with the empirical frequency of accidental forks when we consider as propagation delay the time it takes for a block to reach 50% of nodes in the peer-to-peer network. This would indicate that Bitcoin miners are operating in fair competition, but that most of them have better connectivity than the majority of nodes in the network, thus suggesting a core-periphery structure, where mining pools reside in the core. Note, however, that as most of the miners we consider are actually mining pools, the results would also suggest that cooperation between miners might still be occurring within pools, either through co-location to reduce network delay or by other forms of coordination, as the empirical fork rate is consistent with the model assumption of no intra-pool latency and competition. Beyond its empirical validation, the model formally describes the positive influence of block propagation delay and total mining power over fork rate. It also suggests that a stronger concentration of hash rates—reflected by the fat-tailedness of the distribution—reduces the expected fork rate.

Moreover, we confirm a tendency toward hash rate concentration over time (Table S1a) which is a potential source of concern. Indeed while this would imply lower fork rates and a more efficient consensus mechanism, it introduces security risks that could make the network more vulnerable. Further research is needed to fully explore this trade-off, and we envision our results and methodology to provide a robust mathematical setting for future works in this direction.

Finally, our model assumes that miners are all in fair competition, but a similar framework could also be used to study the impact of selfish mining and other block withholding attacks. While we do not explore this direction in this article, introducing malicious miners is feasible and could be used as a null model for detection of block withholding attacks, in the spirit of (27).

## Methods and data collection

The mining dynamics in the Bitcoin network naturally changes as miners join and abandon the mining activity. However, we need to assume a period of a certain number of consecutive blocks to be approximately stationary to estimate hash rates. By default, we consider a period to be equivalent to one difficulty epoch—a difficulty adjustment cycle of 2,016 blocks (∼2 weeks). The desired statistics on blocks and miners are then computed period-wise (see Table S1 for excerpts). We additionally examine alternative specifications in which a period spans 4, 5, or 10 difficulty epochs. Whilst longer windows make the stationarity assumption more difficult to justify, they improve coverage of small miners and stabilize fork-rate estimates, since both a small miner producing a block and the occurrence of a fork are low-probability events. Full summary statistics are provided in Empirical and fitted distributions of hash rates.xlsx in the Supplementary Data.

### Block propagation delays

We obtain propagation times from the Decentralized Systems and Network Services Research Group at KASTEL^f^—a reputable source often used by related academic works (9), which report the empirical block propagation times to reach 50, 90, and 99% of the Bitcoin network approximately every hour starting from July 2015.

### Forks

The public GitHub repository bitcoin-data/stale-blocks contains up-to-date, crowd-sourced information on stale blocks and their respective block number. We complement this dataset with anecdotal fork data published by Neudecker et al. (9) and orphan block data from Blockchain.com. We then compute the fork rate at a given period as the number of block heights at which at least one stale or orphan block appeared, divided by the total number of blocks in that period. Existing literature also reports similar numbers; for example, (33) estimates a fork rate of 0.41% in February 2016, in line with our source which shows fork rates to fluctuate between 0.35 and 0.45% in the same period (see Supplementary data).

### Miners

In our framework, a miner can be an independent miner or can be a mining pool. In case of the latter, we assume that there is no propagation delay among the members within the same mining pool. To obtain empirical miner information, we fetch coinbase transaction information including block_time, block_timestamp and output.addresses of all the blocks in the Bitcoin blockchain from the public dataset crypto_bitcoin on Google BigQuery. We then identify blocks mined by mining pools mainly using block-miner data from cloverpool.com, complemented by looking up coinbase transactions’ output addresses on the mining pool dictionary data from Blockchain.com. For the remaining unidentified mining addresses, we use a simple heuristic to cluster: if two addresses ever appear in the same coinbase transaction, we consider them to belong to the same miner (which can be a mining pool). The total number of miners *N* is thus estimated as the number of those mining clusters. Figure 2 illustrates the distribution of blocks mined among miners throughout time.

### Hash rates

We retrieve the daily average total hash rate, that is, the average number of H/s that can be generated by the aggregate network on a specific day, on the Bitcoin network through Coin Metrics.^g^ We additionally convert the value of bits of each block fetched from crypto_bitcoin dataset to the level of mining difficulty (Fig. S1); the resultant difficulty value describes the expected number of hashes needed to mine one block. For each period (by default equivalent to a difficulty epoch), the normalized total hash rate *Λ* (in blocks/second) is thus computed as the mean ratio between total hash rate (in H/s) and difficulty (in hashes/block). For each period, the calculated *Λ* value equals approximately the reciprocal of block time $t_{\min}$ (SI Table S1a), which is in accordance with our model description (4).

#### Hash rate distribution estimation

For each period, we count the number of blocks $\left\{b_{i}\right\}$ mined by each distinct miner i∈{1,2,…,N}, where we recall $\sum_{i}^{N}b_{i}=B=2,016$ (one difficulty epoch) in the default scenario. Miners’ hash rates $\left\{\lambda_{i}\right\}$ are then estimated as $\left\{\frac{b_{i}\Lambda }{B}\right\}$. We can then apply the method of moments to estimate the parameters the null distributions presented in SI Appendix, “Null distributions for i.i.d. $\left\{\lambda_{i}\right\}$,” from the empirical mean $m=\frac{\Lambda }{N}$ and standard deviation $s=\sqrt{\mathrm{Var}\frac{b_{i}\Lambda }{B}}$; specifically, the rate parameter *r* of Exp(r), *μ* and *σ* of $\text{LN}(\mu ,\sigma^{2})$, as well as *α* and *β* of truncated power law TPL(α,β) (SI Appendix, “Null distributions for i.i.d. $\left\{\lambda_{i}\right\}$”) are estimated as follows:


(15) $$\begin{array}{rl} r & =\frac{1}{m},\\ \sigma & =\sqrt{\ln\;[1+(s/m{)}^{2}]},\\ \mu & =\ln\;m-\frac{s^{2}}{2},\\ \alpha & =1-{\left(\frac{m}{s}\right)}^{2},\\ \beta & =\frac{m}{s^{2}}. \end{array}$$

We display the estimated parameters in Table S1b.

## Supplementary Material

pgag135_Supplementary_Data

## Data Availability

All code used to fetch and process the raw data are publicly accessible at https://github.com/xujiahuayz/fork.

## Notes

a Source: https://www.blockchain.com/explorer/charts/pools, accessed 18 November 2024.

b See also BBC coverage https://www.bbc.co.uk/news/technology-58896545, accessed 2026 January 28.

c This considers the existence of a few “zero minors”—small miners who fail to produce any block in one difficulty epoch—that we know exist based on observations from longer time windows. See SI Appendix, “Different number of ‘zero miners’” for related discussion.

d Cambridge Centre for Alternative Finance. Cambridge Bitcoin Electricity Consumption Index. Available at: https://ccaf.io/cbnsi/cbeci, accessed 2025-05-21. Licensed under CC BY-NC-SA 4.0.

e
https://grid.iamkate.com/, accessed 2026 January 28.

f
https://www.dsn.kastel.kit.edu/bitcoin/data/invstat.gpd, accessed 2024 October 13.

g
https://raw.githubusercontent.com/coinmetrics/data/refs/heads/master/csv/btc.csv, accessed 2026 January 28.

## Preprints

This manuscript was posted on a preprint: https://arxiv.org/abs/2411.10249.

## References

[1] Nakamoto S . 2008. Bitcoin: a peer-to-peer electronic cash system. https://assets.pubpub.org/d8wct41f/31611263538139.pdf.

[2] Lilly GM . 2004. Device for and method of one-way cryptographic hashing.

[3] Arthur WB . Increasing returns and path dependence in the economy. University of Michigan Press, 1994.

[4] Avarikioti G, Käappeli L, Yuyi W, Wattenhofer R. Bitcoin security under temporary dishonest majority. In: *Financial Cryptography and Data Security 2019 Proceedings*. Springer Nature, 2019, p. 1–17.

[5] Lehar A, et al 2025. Market power and the Bitcoin protocol.

[6] Decker C, Wattenhofer R. Information propagation in the Bitcoin network. In: *IEEE P2P Proceedings*. IEEE; 2013. p. 1–10. 10.1109/P2P.2013.6688704

[7] Eyal I, Sirer EG. 2018. Majority is not enough. Commun ACM. 61:95–102. 10.1145/3212998

[8] Fadda E, He J, Tessone CJ, Barucca P. 2022. Consensus formation on heterogeneous networks. EPJ Data Sci. 11:34. 10.1140/epjds/s13688-022-00347-5

[9] Neudecker T, Hartenstein H. Short paper: an empirical analysis of blockchain forks in Bitcoin. In: Financial cryptography and data security. 2019. p. 84–92. 10.1007/978-3-030-32101-7_6

[10] Shahsavari Y, Zhang K, Talhi C. A theoretical model for fork analysis in the bitcoin network. In: *IEEE International Conference on Blockchain*. Institute of Electrical and Electronics Engineers Inc; 2019. p. 237–244. 10.1109/BLOCKCHAIN.2019.00038

[11] Misic VB, Misic J, Chang X. On forks and fork characteristics in a Bitcoin-like distribution network. In: *IEEE International Conference on Blockchain*. Institute of Electrical and Electronics Engineers Inc; 2019. p. 212–219. 10.1109/BLOCKCHAIN.2019.00035

[12] Tessone C, Tasca P, Iannelli F. 2021. Stochastic modelling of Blockchain consensus [preprint], arXiv 2021, arXiv:2106.06465v1, 10.48550/arXiv.2106.06465

[13] Liu Q, Xu Y, Cao B, Zhang L, Peng M. 2021. Unintentional forking analysis in wireless Blockchain networks. Digit Commun Netw. 7:335–341. 10.1016/j.dcan.2020.12.005

[14] Alzayat M, et al 2021. Modeling coordinated vs. P2P mining: an analysis of inefficiency and inequality in proof-of-work Blockchains [preprint], arXiv 2021, arXiv:2106.02970v1.

[15] Cao T, Decouchant J, Yu J, Esteves-Verissimo P. Characterizing the impact of network delay on Bitcoin mining. In: *40th International Symposium on Reliable Distributed Systems*. Volume 2021-September. IEEE; 2021 p. 109–119. 10.1109/SRDS53918.2021.00020

[16] Long S, Basu S, Sirer EG. 2022. Measuring miner decentralization in proof-of-work Blockchains [preprint], arXiv 2022, arXiv:2203.16058v1.

[17] Mao Y, Venkatakrishnan SB. 2023. Less is more: understanding network bias in proof-of-work Blockchains. Mathematics. 11:4741. 10.3390/MATH11234741

[18] Saad M, Anwar A, Ravi S, Mohaisen D. Revisiting Nakamoto consensus in asynchronous networks: a comprehensive analysis of Bitcoin safety and chain quality. In: *Proceedings of the 2021 ACM SIGSAC Conference on Computer and Communications Security (CCS)*. ACM; 2021. p. 987–1001. 10.1145/3460120.3484561

[19] Saad M, Chen S, Mohaisen D. Syncattack: double-spending in Bitcoin without mining power. In: *Proceedings of the 2021 ACM SIGSAC Conference on Computer and Communications Security (CCS)*. ACM; 2021. p. 1667–1682. 10.1145/3460120.3484568

[20] Saad M, Chen S, Mohaisen D. Root cause analyses for the deteriorating Bitcoin network synchronization. In: *Proceedings of the 41st IEEE International Conference on Distributed Computing Systems (ICDCS)*. IEEE; 2021. p. 239–249. 10.1109/ICDCS51616.2021.00031

[21] Saad M, Cook V, Nguyen L, Thai MT, Mohaisen D. 2022. Exploring partitioning attacks on the Bitcoin network. IEEE ACM Trans Netw. 30:202–216. 10.1109/TNET.2021.3105604

[22] Eyal I, Sirer EG. Majority is not enough: Bitcoin mining is vulnerable. In: *International Conference on Financial Cryptography and Data Security*. Springer Nature, 2014. p. 436–454.

[23] Nayak K, Kumar S, Miller A, Shi E. Stubborn mining: generalizing selfish mining and combining with an eclipse attack. In: *Proceedings—2016 IEEE European Symposium on Security and Privacy, EURO S and P 2016*. IEEE, 2016. p. 305–320. 10.1109/EUROSP.2016.32

[24] Liu H, Du R, Ruan N, Jia W. On the strategy and behavior of Bitcoin mining with N-attackers. In: *ASIACCS 2018 - Proceedings of the 2018 ACM Asia Conference on Computer and Communications Security*. Vol. 12. ACM, 2018. p. 357–368. 10.1145/3196494.3196512

[25] Sarenche R, Nikova S, Preneel B. 2025. Mining power destruction attacks in the presence of petty-compliant mining pools [preprint], arXiv 2025, arXiv:2502.07410v1.

[26] Xiao Y, Zhang N, Lou W, Hou YT. Modeling the impact of network connectivity on consensus security of proof-of-work Blockchain. In: *IEEE INFOCOM*, volume 2020-July. IEEE; 2020. p. 1648–1657. 10.1109/INFOCOM41043.2020.9155451

[27] Li SN, Campajola C, Tessone CJ. 2024. Statistical detection of selfish mining in proof-of-work Blockchain systems. Sci Rep. 14:6251–6251. 38491037 10.1038/s41598-024-55348-3 PMC10943236

[28] Bahrani M, Weinberg SM. 2024. Undetectable selfish mining. 10.1145/3670865.3673485

[29] Yaish A, Stern G, Zohar A. 2023. Uncle maker: (time)stamping out the competition in ethereum. 10.1145/3576915.3616674

[30] Anas Imtiaz M, Starobinski D, Trachtenberg A, Younis N. 2021. Churn in the Bitcoin network. IEEE Trans Netw Serv Manage. 18:1598–1615.

[31] Campajola C, et al 2022. The evolution of centralisation on cryptocurrency platforms [preprint], arXiv 2022, arXiv:2206.05081v2.

[32] Makarov I, Schoar A. 2021. Igor makarov and antoinette schoar: blockchain analysis of the Bitcoin market. *SSRN 3942181*. 10.3386/W29396

[33] Gervais A, et al On the security and performance of proof of work Blockchains. In: *Proceedings of the 2016 ACM SIGSAC Conference on Computer and Communications Security*, volume 24-28-Octo, New York, NY, USA. ACM; 2016. p. 3–16. 10.1145/2976749.2978341

