# Supplementary material for: How the interplay between power concentration, competition, and propagation affects the resource efficiency of distributed ledgers
Source: PNAS Nexus. 2026 May 26;5(5):pgag135. doi: 10.1093/pnasnexus/pgag135 (PMC13203942; doi:10.1093/pnasnexus/pgag135)
Supplement: pgag135_Supplementary_Data [file pgag135_supplementary_data.zip › BCX_Supplementary_Material.pdf]

Supporting Information for

How the interplay between power concentration, competition, and propagation affects the resource efficiency of distributed ledgers

Paolo Barucca, Carlo Campajola and Jiahua Xu

Paolo Barucca, E-mail: p.barucca@ucl.ac.uk;
Carlo Campajola, E-mail: c.campajola@ucl.ac.uk;
Jiahua Xu, E-mail: jiahua.xu@ucl.ac.uk.

This PDF file includes:

Supporting text
Figs. S1 to S5
Table S1
SI References

Supporting Information Text

Derivation of Eq. 10 in *Research Article*

Let $CC(\Delta) = \int_D p(\{\lambda_i\}) \left(\frac{\sum_i^N \frac{\lambda_i}{e^{\Delta \sum_{j\neq i} \lambda_j}}}{\sum_i^N \lambda_i} \right) d\{\lambda_i\}$ denotes the complementary cumulative function representing the probability of no fork. We now consider the convenient integral expression for the denominator: $\frac{1}{\sum_i^N \lambda_i} = \int_0^\infty e^{-x \sum_i^N \lambda_i} dx$. Thus,

$$
\begin{aligned}
CC(\Delta) &= \int_D p(\{\lambda_i\}) \left(\int_0^\infty e^{-x\sum_i^N \lambda_i} dx \sum_i^N \frac{\lambda_i}{e^{\Delta \sum_{j\neq i} \lambda_j}} \right) d\{\lambda_i\} \\
&= \int_0^\infty dx \int_D p(\{\lambda_i\}) \left(\sum_i^N \frac{\lambda_i}{e^{\Delta \sum_{j\neq i} \lambda_j + x \sum_i^N \lambda_i}} \right) d\{\lambda_i\} \\
&= \int_0^\infty dx \underbrace{\int_D p(\{\lambda_i\}) \left(\sum_i^N \frac{\lambda_i}{e^{x\lambda_i}} \prod_{j\neq i} \frac{1}{e^{(\Delta+x)\lambda_j}} \right) d\{\lambda_i\}}_{\mathcal{A}}.
\end{aligned}
$$

When $\lambda_i > 0$ for all i and $\{\lambda_i\}$ are independent, we can express $\mathcal{A}$ as

$$
\mathcal{A} = \int_0^\infty \cdots \int_0^\infty \left(\sum_i^N \frac{\lambda_i p(\lambda_i) d\lambda_i}{e^{x\lambda_i}} \prod_{j\neq i} \frac{p(\lambda_j) d\lambda_j}{e^{(\Delta+x)\lambda_j}} \right) = \sum_i^N \left(\int_0^\infty \frac{\lambda_i p(\lambda_i) d\lambda_i}{e^{x\lambda_i}} \prod_{j\neq i} \int_0^\infty \frac{p(\lambda_j) d\lambda_j}{e^{(\Delta+x)\lambda_j}} \right),
$$

hence Eq. 10 in *Research Article*.

Considered distributions for $\{\lambda_i\}$

Empirical distribution. Give the number of blocks mined by miner i $\{b_i\}$ in a certain set of blocks of size $B = \sum_i b_i$, if we use frequentist inference, then we can approximate λ_i as

$$
\lambda_i = \frac{b_i \cdot \Lambda}{B}, \qquad [1]
$$

under which *HHI* can be computed as

$$
HHI = \sum_i^N \left(\frac{b_i}{B} \right)^2, \qquad [2]
$$

and fork rate can be calculated as

$$
C(\Delta_0 | \{\lambda_i = \frac{b_i \cdot \Lambda}{B}\}) = 1 - \sum_i^N \frac{\frac{b_i}{B}}{e^{\Delta_0 \Lambda \left(1 - \frac{b_i}{B}\right)}}. \qquad [3]
$$

Semi-empirical distributions. Under a Bayesian framework in order to, we can account for the uncertainty on the true value of the hash rates conditioned over a given measurement $\{b_i\}$. The likelihood $p(b|\lambda)$ of observing b blocks being mined by a miner with hash rate λ follows a Gaussian distribution $p(b|\lambda) = \frac{e^{-\frac{(b-\gamma\cdot\lambda)^2}{2\gamma\cdot\lambda}}}{\sqrt{2\pi\gamma\cdot\lambda}}$, which is in principle unbounded over b but effectively centered around the value $\gamma \cdot \lambda$, where $\gamma = \frac{B}{\Lambda}$. Applying Bayes theorem and assuming a flat prior, we can introduce the empirical distribution over λ conditioned on a given number of mined blocks:

$$
p(\lambda|b) = \frac{e^{-\frac{(b-\gamma\cdot\lambda)^2}{2\gamma\cdot\lambda}}}{\sqrt{2\pi\lambda/\gamma}}. \qquad [4]
$$

***INID* $\{\lambda_i\}$.** Assuming $\{\lambda_i\}$ are independent but not necessarily identically distributed (INID), we plug Eq. 4 in Eq. 10 in *Research Article* to obtain

$$
C(\Delta_0) = 1 - \int_0^\infty \frac{\sum_i^N \left(1 + b_i \cdot \sqrt{1+\frac{2x}{\gamma}}\right) e^{B\left(1 - \frac{2(\Delta_0+x)}{\gamma}\right) + b_i \left(\sqrt{1+\frac{2(\Delta_0+x)}{\gamma}} - \sqrt{1+\frac{2x}{\gamma}} \right)}}{\gamma \left(\sqrt{1+\frac{2x}{\gamma}}\right)^3 \left(\sqrt{1+\frac{2(\Delta_0+x)}{\gamma}}\right)^{N-1}} dx. \qquad [5]
$$

***i.i.d.* $\{\lambda_i\}$.** In the even more specific case of λ_i being i.i.d., we can introduce the semi-empirical marginal distribution for λ by averaging $p(\lambda|\{b_i\})$ over $\{b_i\}$, finding

$$p(\lambda|\{b_i\}) = \frac{1}{N}\sum_{i}^{N} \frac{e^{-\frac{(b_i-\gamma\cdot\lambda)^2}{2\gamma\cdot\lambda}}}{\sqrt{2\pi\lambda/\gamma}}. \qquad [6]$$

And so, plugging Eq. 6 into Eq. 11 in *Research Article*, we arrive at

$$C(\Delta_0) = 1 - \int_0^\infty \frac{\sum_i^N \left(1+b_i\cdot\sqrt{1+\frac{2x}{\gamma}}\right) e^{b_i\left(1-\sqrt{1+\frac{2x}{\gamma}}\right)}}{\gamma\left(\sqrt{1+\frac{2x}{\gamma}}\right)^3} \left[\frac{\sum_i^N e^{b_i\left(1-\sqrt{1+\frac{2(\Delta_0+x)}{\gamma}}\right)}}{N\sqrt{1+\frac{2(\Delta_0+x)}{\gamma}}}\right]^{N-1} dx. \qquad [7]$$

Null distributions for i.i.d. $\{\lambda_i\}$. As an alternative approach to the Bayesian setting described above, we also consider 3 commonly used null distributions for i.i.d. $\{\lambda_i\}$, assuming they can be distributed according to an exponential, log-normal or truncated power law distribution. This i.i.d. hypothesis refers to the Bayesian prior on the distribution of hash rates per miner, yet that does not imply a i.i.d. distribution for the corresponding mining times, which are sampled conditioned on the specific hash rate of each miner.

Exponential. We select an exponential distribution for its parsimony, as the distribution can be characterized simply by one rate parameter r. For $\lambda_i \sim \text{Exp}(r)\ \forall i$, we plug the density function

$$p(\lambda_i) = re^{-r\lambda_i}, \qquad [8]$$

into Eq. 11 *Research Article* and get

$$C(\Delta_0) = 1 - N\int_0^\infty \left[\left(\int_0^\infty \frac{\lambda r d\lambda}{e^{x\lambda+r\lambda}}\right)\left(\int_0^\infty \frac{r d\lambda}{e^{(\Delta_0+x)\lambda+r\lambda}}\right)^{N-1}\right] dx \qquad [9]$$

$$= 1 - Nr^N\int_0^\infty \frac{dx}{(r+x)^2(\Delta_0+r+x)^{N-1}}. \qquad [10]$$

Log-normal. A log-normal distribution is frequently observed in systems characterised by unbounded multiplicative (or proportional) growth (1), and there is a large body of literature supporting the view that blockchain consensus protocols are strongly affected by this accumulation dynamics (2–5). For $\lambda_i \sim \text{LN}(\mu, \sigma^2)\ \forall i$, we integrate numerically after plugging its density function

$$p(\lambda_i) = \frac{e^{\frac{-(\ln\lambda_i-\mu)^2}{2\sigma^2}}}{\lambda_i\sigma\sqrt{2\pi}} \qquad [11]$$

into Eq. 11 *Research Article.*

Truncated power law. A truncated power law distribution also known as power law distribution with exponential cut-off, typically emerges in complex systems where proportional growth effects get capped by some natural scale or limit (6). In the specific context of mining, the cap we would see is given by the fact that the total hashrate sums to Λ, meaning that each individual miner cannot have a hashrate larger than that in a given sample. This limitation results in a truncation of the power law at the upper end, which is generally captured by multiplying the power law function with a decreasing exponential. As above, for $\lambda_i \sim \text{TPL}(\alpha, \beta)\ \forall i$, we can plug the density function

$$p(\lambda_i) = \frac{\beta^{1-\alpha}}{\Gamma(1-\alpha)\cdot\lambda_i^\alpha\cdot e^{\beta\lambda_i}} \qquad [12]$$

into Eq. 11 *Research Article* and arrive at

$$C(\Delta_0) = 1 - N(1-\alpha)\cdot\beta^{N(1-\alpha)}\int_0^\infty \frac{dx}{(\beta+x)^{2-\alpha}\cdot(\Delta_0+\beta+x)^{(N-1)(1-\alpha)}}. \qquad [13]$$

For succinctness we omit the derivation steps. Note that the truncated power law distribution can be deemed as a generalization of the exponential distribution. Specifically, when $\beta = r$ and $\alpha = 0$, $\text{TPL}(\alpha, \beta)$ can be reduced to $\text{Exp}(r)$.

Different number of "zero miners". When estimating hash rates from mined block frequencies, we may need to consider the possibility of existent miners that have not been able to mine a block in the period, who we term as "zero miners". In general, assuming a fair sampling, the probability of mining should be simply proportional to the hash rate and the inability of a miner to mine in a long time horizon should reflect a very low hash rate. Nevertheless, some miners with non-negligible hash power may, by chance, produce no blocks within a finite window, with this probability decreasing as the window length increases. In Fig. S2, we depict the scenarios of different numbers of zero miners being added to the miners observed from our last sample period (Fig. S5**F**). For a given number of zero miners, we refit the distributions under the i.i.d. assumption. The fork rates calculated with the refitted distribution with different numbers of zero miners N_0 are depicted in Fig. S3**B**-Fig. S3**E**.

We additionally check the scenario with miner hash rates following independent, but non-identical distributions applying the semi-empirical approach. The distribution of a zero miner's hash rate is essentially the posterior distribution conditioned on zero blocks, which can be written out by plugging $b = 0$ into Eq. 4: $p(\lambda|0) = \frac{e^{-\frac{\gamma \cdot \lambda}{2}}}{\sqrt{2\pi\lambda/\gamma}}$. The corresponding result is illustrated in Fig. S3**A**. Overall, the effect of "zero miners" is almost negligible regardless of the distribution type used or block propagation delay.

Simulations

To validate our analytical derivations, we conduct simulations within a synthetic blockchain environment. For each round, we initiate the environment with $N \geq 2$ miners, whose hash powers $\{\lambda_i\}_{i=1,2,\ldots,N}$ are randomly sampled from a null distribution, To simulate the mining process, we generate for each miner i a mining time t_i following an exponential distribution with rate λ_i, i.e., $t_i \sim \text{Exp}(\lambda_i)$, mimicking the Poisson arrival pattern of mined blocks (Modeling consensus propagation in a heterogeneous network, in *Research Article*). We calculate the time difference Δ between the two fastest miners for each round. A fork is deemed to have occurred if Δ is below the pre-specified block propagation time Δ_0; otherwise, the round concludes with no fork. For a given set of hyper-parameters, i.e. the number of miners N, mean m and standard deviation s of the hash power distribution, and Δ_0, we iterate for n rounds—where n is sufficiently large ($n = 10^7$ in our experiments)—and count the number of rounds with a fork denoted as n_{fork}. The simulated fork rate is then calculated as the fraction of simulations where a fork occurred, $\frac{n_{\text{fork}}}{n}$.

In Fig. S4, we superimpose the analytical results (thin, opaque curves) on the simulated output (thick, transparent curves) for the null distributions—exponential $\text{Exp}(r)$, log-normal $\text{LN}(\mu, \sigma^2)$ and truncated power law $\text{TPL}(\alpha, \beta)$. The distribution parameters are calculated as Eq. 15 in *Research Article* at $m = 5 \times 10^{-5}$ and $s = 1 \times 10^{-4}$, approximately the same level of the mean and standard deviation of hash rates estimated from our last few sample periods (Table S1). We also compute the fork rate using the semi-empirical method under the i.i.d. assumption. Specifically, we assume each miner's hash rate is randomly sampled from the same distribution with the PDF estimated following Eq. 6 using the numbers of blocks mined from the 35 miners and the total hash rate $\Lambda = 0.0017$ [s^{-1}]. The results are plotted in dashed red curves.

Fig. S4 shows that our analytical result correctly predicts the simulation with remarkable accuracy regardless of the underlying distribution, and that, holding hash rate mean m and standard deviation s constant, the fork rate increases with the number of participating miners and the block propagation delay, as could be expected.

Derivation of Eq. 12 and Eq. 13 in *Research Article*. We write out the expected value of $\sum_{i\neq k}\lambda_i$:

$$\begin{aligned}
\mathbb{E}\left(\sum_{i\neq k}\lambda_i\right) &= \int_D p(\{\lambda_i\}|k \text{ wins})\left(\sum_{i\neq k}\lambda_i\right)d\{\lambda_i\}\\
&= \int_D \frac{p(k \text{ wins}|\{\lambda_i\})\cdot p(\{\lambda_i\})}{p(k \text{ wins})}\left(\sum_{i\neq k}\lambda_i\right)d\{\lambda_i\}\\
&= \int_D \frac{\frac{\lambda_k}{\sum_i^N \lambda_i}\cdot p(\{\lambda_i\})}{\frac{1}{N}}\left(\sum_{i\neq k}\lambda_i\right)d\{\lambda_i\}\\
&= N\int_D \lambda_k\left(\sum_{i\neq k}\lambda_i\right)\left(\int_0^\infty e^{-x\sum_i^N\lambda_i}dx\right)\cdot p(\{\lambda_i\})d\{\lambda_i\}\\
&= N\int_0^\infty dx\int_D \frac{\lambda_k\left(\sum_{i\neq k}\lambda_i\right)}{e^{x\sum_i^N\lambda_i}}\cdot p(\{\lambda_i\})d\{\lambda_i\}\\
&= N\int_0^\infty dx\int_D \lambda_k\left(\sum_{i\neq k}\lambda_i\right)\cdot\prod_i^N\frac{p(\{\lambda_i\})}{e^{x\lambda_i}}d\{\lambda_i\}\\
&= N\int_0^\infty dx\underbrace{\sum_{i\neq k}\int_D \lambda_k\lambda_i\cdot\prod_i^N\frac{1}{e^{x\lambda_i}}\cdot p(\{\lambda_i\})d\{\lambda_i\}}_{\mathcal{B}},
\end{aligned} \qquad [14]$$

hence Eq. 12 in *Research Article*.

Assume for all i, λ_i are independent, we can express $\mathcal{B}$ as

$$\begin{aligned}
\mathcal{B} &= \sum_{i\neq k}\left[\int_0^\infty ...\int_0^\infty \lambda_k\lambda_i\prod_i^N\frac{p(\lambda_i)d\lambda_i}{e^{x\lambda_i}}\right]\\
&= \sum_{i\neq k}\left[\int_0^\infty\frac{\lambda_i p(\lambda_i)d\lambda_i}{e^{x\lambda_i}}\cdot\int_0^\infty\frac{\lambda_k p(\lambda_k)d\lambda_k}{e^{x\lambda_k}}\cdot\prod_{j\neq i,k}^N\int_0^\infty\frac{p(\lambda_j)d\lambda_j}{e^{x\lambda_j}}\right]\\
&= (N-1)\left[\left(\int_0^\infty\frac{\lambda p(\lambda)d\lambda}{e^{x\lambda}}\right)^2\left(\int_0^\infty\frac{p(\lambda)d\lambda}{e^{x\lambda}}\right)^{N-2}\right]
\end{aligned} \qquad [15]$$

hence Eq. 13 in *Research Article*.

Table S1. Empirical and fitted distributions of hash rates for selected periods, each with a fixed-length window of 2,016 blocks. For complete summary statistics covering the entire sample, see `Empirical and fitted distributions of hash rates.xlxs` in Supplementary data.

(a) Empirical data

period of blocks		propagation time						empirical miner hash rate						
start #	start time	50% [s]	90% [s]	99% [s]	block time t_{min} [s]	fork rate [%]	miners N	$\sum$(hash rate) Λ [block/s]	mean m [block/s]	std s [block/s]	skewness	kurtosis	hhi	max share [%]
364896	2015-07-11	8.89	18.42	27.74	586.3	0.992	28	0.00171	0.000061	0.000097	1.95	2.85	0.12	19.39
385056	2015-11-24	7.36	18.84	28.21	551.7	0.893	18	0.00182	0.000101	0.000135	1.55	1.40	0.15	22.87
405216	2016-04-01	6.49	16.54	27.96	560.4	0.298	20	0.00179	0.000090	0.000143	2.09	3.73	0.17	28.03
425376	2016-08-15	4.41	12.93	26.24	591.4	0.149	18	0.00170	0.000095	0.000103	0.94	-0.29	0.12	19.05
445536	2016-12-28	3.32	10.68	24.33	566.1	0.149	27	0.00178	0.000066	0.000090	1.91	3.61	0.10	19.64
465696	2017-05-10	3.17	13.11	26.47	564.0	0.397	29	0.00178	0.000061	0.000074	1.49	2.33	0.08	16.91
485856	2017-09-18	0.70	5.00	16.25	589.2	0.000	28	0.00172	0.000062	0.000080	1.58	2.18	0.09	17.96
506016	2018-01-25	0.85	8.27	21.53	543.4	0.099	21	0.00187	0.000089	0.000126	1.86	2.98	0.14	24.70
526176	2018-06-05	0.46	2.62	14.13	584.0	0.050	25	0.00173	0.000069	0.000108	2.58	7.80	0.13	27.58
546336	2018-10-18	0.47	2.34	15.35	600.5	0.000	34	0.00168	0.000049	0.000080	1.80	2.13	0.10	17.41
566496	2019-03-10	0.43	2.50	16.05	570.8	0.000	32	0.00177	0.000055	0.000076	1.49	1.05	0.09	15.23
586656	2019-07-23	0.39	2.53	17.55	542.0	0.000	28	0.00185	0.000066	0.000098	1.62	1.84	0.11	19.44
606816	2019-12-05	0.39	2.02	14.31	596.9	0.000	26	0.00168	0.000065	0.000086	1.65	1.88	0.10	16.82
626976	2020-04-21	0.61	3.87	16.94	594.6	0.000	20	0.00168	0.000084	0.000101	1.30	0.63	0.12	18.60
647136	2020-09-07	0.43	2.50	14.94	539.5	0.050	20	0.00186	0.000093	0.000098	1.02	0.01	0.10	16.91
667296	2021-01-23	0.76	4.41	19.07	583.0	0.050	24	0.00172	0.000072	0.000085	1.08	-0.11	0.10	16.17
687456	2021-06-13	0.56	3.10	16.18	832.9	0.000	24	0.00124	0.000052	0.000069	1.33	0.59	0.11	18.60
707616	2021-10-31	0.37	1.56	13.60	573.7	0.050	17	0.00175	0.000103	0.000108	0.69	-1.10	0.12	17.66
727776	2022-03-17	0.36	1.32	13.72	576.3	0.000	15	0.00173	0.000115	0.000106	0.45	-1.35	0.12	17.96
747936	2022-08-04	0.34	1.24	11.86	596.8	0.000	17	0.00169	0.000099	0.000120	1.32	1.18	0.14	24.11
768096	2022-12-19	0.35	1.27	11.34	623.4	0.000	16	0.00165	0.000103	0.000139	1.59	1.57	0.17	27.18
788256	2023-05-04	1.66	11.88	27.46	581.5	0.347	23	0.00174	0.000075	0.000137	2.41	5.63	0.18	30.70
808416	2023-09-19	0.90	4.80	22.16	598.0	0.099	18	0.00168	0.000093	0.000145	2.04	3.68	0.18	30.31
828576	2024-02-02	0.97	5.24	20.11	555.3	0.050	18	0.00180	0.000100	0.000164	2.10	3.54	0.20	30.51
848736	2024-06-20	0.83	3.26	18.88	631.9	0.099	18	0.00160	0.000089	0.000138	2.02	3.17	0.18	28.72
868896	2024-11-04	0.87	3.29	16.17	596.8	0.099	22	0.00169	0.000077	0.000137	2.49	6.08	0.18	31.65
889056	2025-03-23	0.54	2.49	11.92	561.7	0.000	24	0.00178	0.000074	0.000133	2.58	6.88	0.17	30.85

(b) Fitted parameters

fitted distributions				
Exp(r)	LN(μ, σ^2)		TPL(α, β)	
r	μ	σ	α	β
16,364	-10.33	1.12	0.60	6,508
9,879	-9.71	1.01	0.44	5,564
11,171	-9.96	1.13	0.61	4,356
10,572	-9.66	0.88	0.16	8,913
15,167	-10.15	1.02	0.46	8,222
16,295	-10.15	0.95	0.32	11,096
16,234	-10.19	0.99	0.40	9,681
11,253	-9.88	1.05	0.50	5,603
14,475	-10.20	1.11	0.59	5,904
20,259	-10.56	1.13	0.62	7,776
18,073	-10.33	1.03	0.47	9,597
15,142	-10.21	1.08	0.55	6,882
15,477	-10.15	1.01	0.43	8,837
11,874	-9.83	0.94	0.30	8,330
10,749	-9.66	0.86	0.10	9,675
13,973	-9.99	0.94	0.29	9,854
19,395	-10.39	1.01	0.44	10,806
9,714	-9.55	0.86	0.10	8,775
8,670	-9.37	0.78	-0.18	10,266
10,084	-9.67	0.95	0.32	6,899
9,708	-9.70	1.02	0.45	5,327
13,246	-10.22	1.21	0.70	4,018
10,695	-9.89	1.11	0.59	4,424
9,989	-9.86	1.14	0.63	3,704
11,281	-9.95	1.11	0.59	4,661
13,031	-10.19	1.19	0.68	4,115
13,485	-10.23	1.20	0.69	4,208

(c) Wasted power

			Log normal		Truncated power law	
average difficulty [THash/block]	average efficiency [J/THash]	total hash power [MW]	wasted hash [block/s]	wasted power [MW]	wasted hash [block/s]	wasted power [MW]
219,371,323	1,055.60	396	0.00153	355	0.00151	351
312,341,965	850.32	484	0.00159	421	0.00157	416
716,621,793	516.60	663	0.00154	570	0.00151	558
933,620,589	278.12	442	0.00152	394	0.00151	392
1,364,461,290	279.20	678	0.00162	616	0.00160	611
2,405,056,672	222.14	951	0.00164	877	0.00164	874
4,739,070,921	201.98	1,651	0.00158	1,511	0.00157	1,502
11,180,131,873	171.50	3,578	0.00164	3,150	0.00162	3,110
21,220,165,903	137.14	5,026	0.00153	4,457	0.00151	4,394
30,850,115,780	110.15	5,703	0.00153	5,193	0.00151	5,142
26,065,690,694	89.81	4,145	0.00163	3,814	0.00162	3,792
38,713,920,146	87.07	6,233	0.00167	5,627	0.00165	5,573
55,305,615,651	72.98	6,780	0.00152	6,151	0.00151	6,108
68,541,889,839	68.32	7,887	0.00150	7,044	0.00149	6,994
74,500,493,293	49.88	6,914	0.00168	6,249	0.00167	6,222
89,436,385,277	66.17	10,165	0.00156	9,246	0.00155	9,198
85,610,685,580	62.83	6,656	0.00111	5,984	0.00110	5,935
93,026,177,712	59.42	9,673	0.00156	8,595	0.00155	8,547
117,908,481,743	54.08	11,033	0.00153	9,787	0.00153	9,748
121,009,279,703	43.06	8,783	0.00148	7,686	0.00146	7,612
151,887,506,496	34.96	8,753	0.00141	7,486	0.00139	7,367
206,182,199,904	36.16	12,947	0.00149	11,134	0.00145	10,832
245,327,979,205	31.24	12,898	0.00143	10,984	0.00140	10,739
324,279,332,132	29.43	17,197	0.00152	14,516	0.00148	14,125
359,382,515,042	25.47	14,608	0.00136	12,439	0.00133	12,161
436,569,869,230	23.88	17,604	0.00145	15,095	0.00141	14,689
488,584,780,017	21.99	19,125	0.00154	16,572	0.00150	16,169

```python
    # Maximum target value (for difficulty 1, i.e., the easiest level)
MAX_TARGET = 0x00000000FFFF0000000000000000000000000000000000000000000000000000
def bits_to_difficulty(bits_hex_str: str) -> float:
    """
    Convert 'bits' field from Bitcoin block header to the expected number of hashes needed to mine a block at this difficulty.
    :param bits_hex_str: Hex string representing the 'bits' field (e.g., '1b00dc31').
    :return: Expected number of hashes needed to find a valid block.
    """
    # Extract exponent (first byte) and coefficient (next three bytes)
    exponent = int(bits_hex_str[:2], 16)
    coefficient = int(bits_hex_str[2:], 16)
    # Calculate the current target value
    target = coefficient * (256 ** (exponent - 3))
    # Difficulty is the ratio of max_target to the current target, scaled by 2^32
    return (MAX_TARGET / target) * (2**32)
```

Fig. S1. Python script to convert `bits` to difficulty.

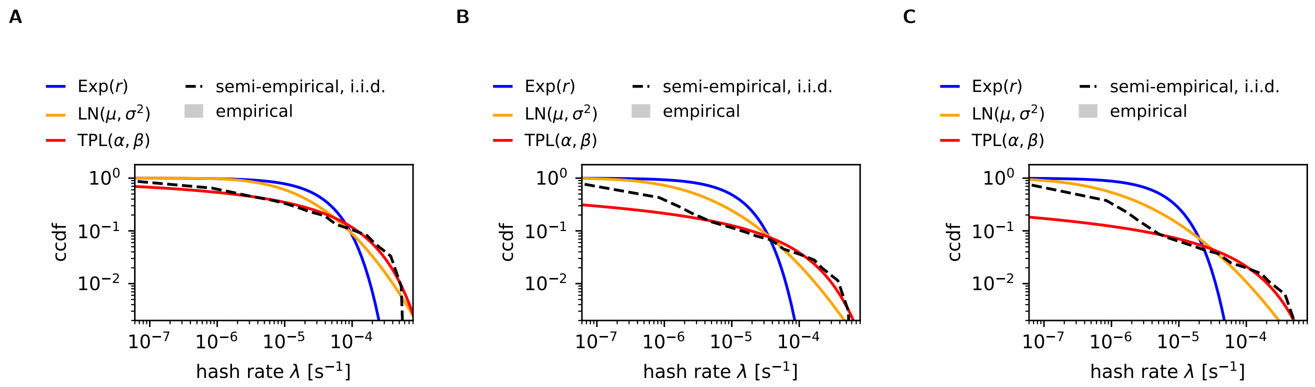

Fig. S2. Complementary cumulative distribution function (ccdf) of hash rates when different numbers of "zero miners" are added to observed miners between block 868896 and 870911 (Fig. S5**F**). **A**: 20 zero miners. **B**: 100 zero miners. **C**: 200 zero miners.

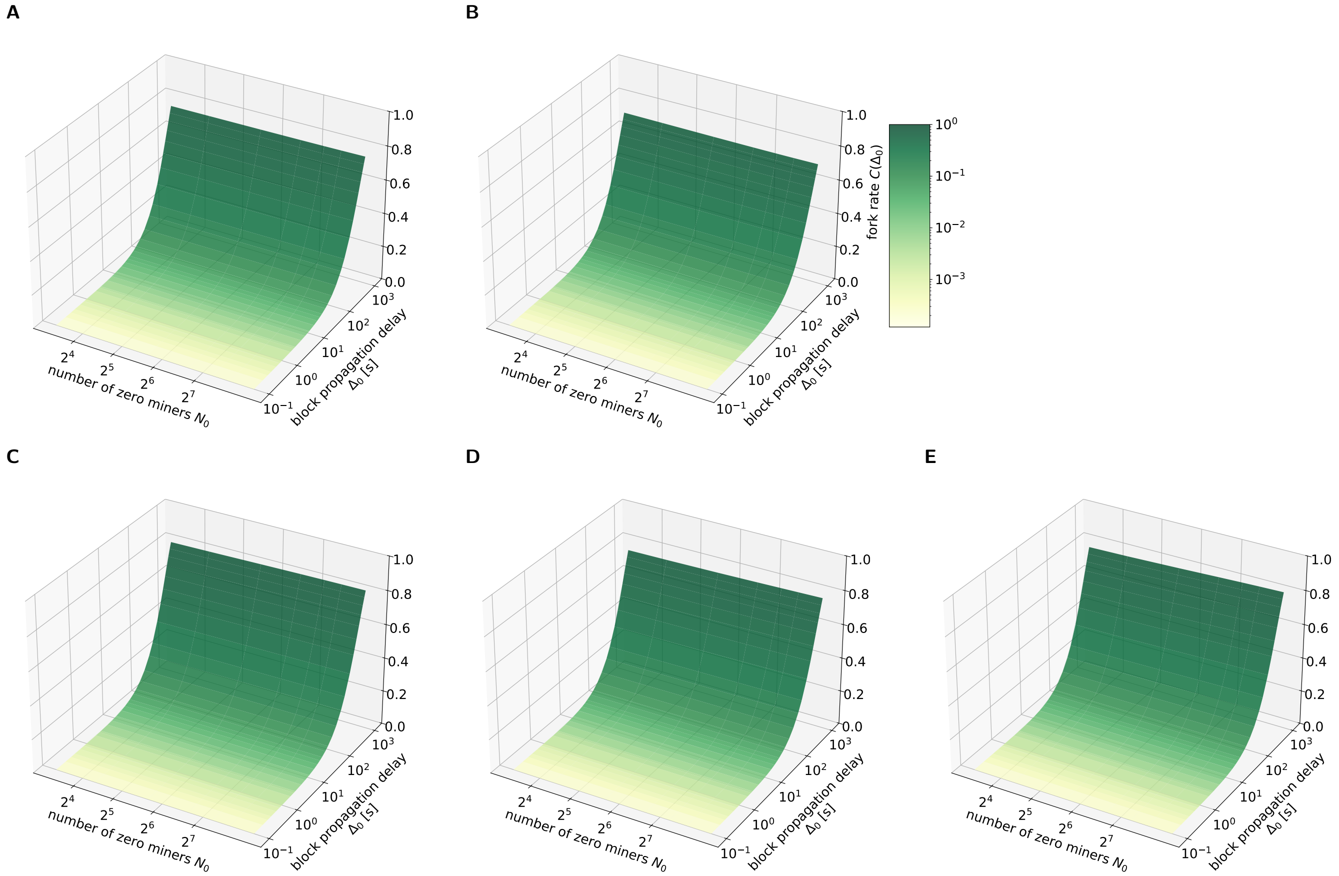

Fig. S3. Fork rates by number of zero miners N_0 and block propagation delay Δ_0 at various given hash-rate distributions. **A**: Semi-emp, independent. **B**: Semi-emp, i.i.d. **C**: Exponential. **D**: Log-normal. **E**: Truncated power law.

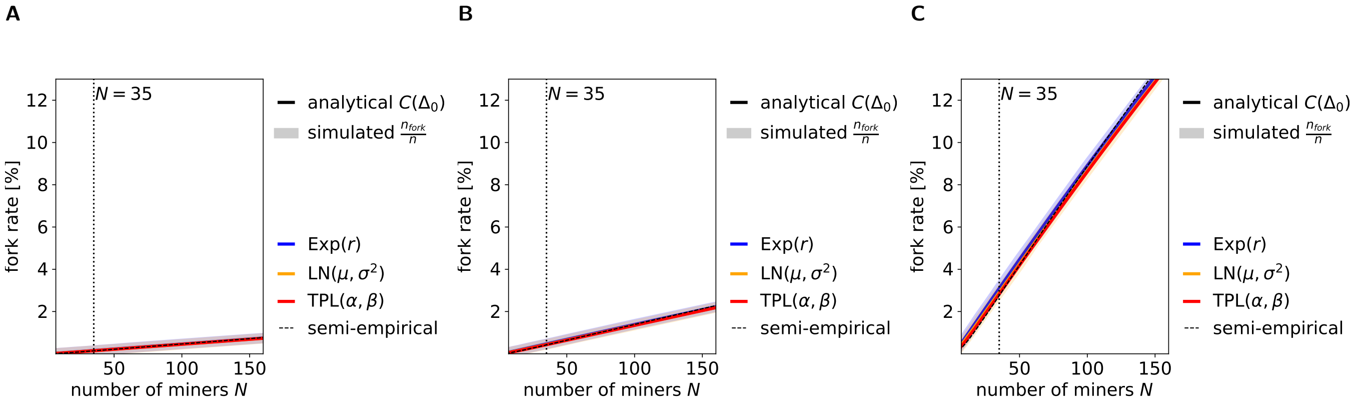

Fig. S4. Fork rates computed from the simulated environment (thick, transparent curves) compared with those calculated analytically (thin, opaque curves) under various block propagation delays, hash rate distributions, and miner numbers. **A**: $\Delta_0 = 1$ [s]. **A**: $\Delta_0 = 3$ [s]. **A**: $\Delta_0 = 20$ [s].

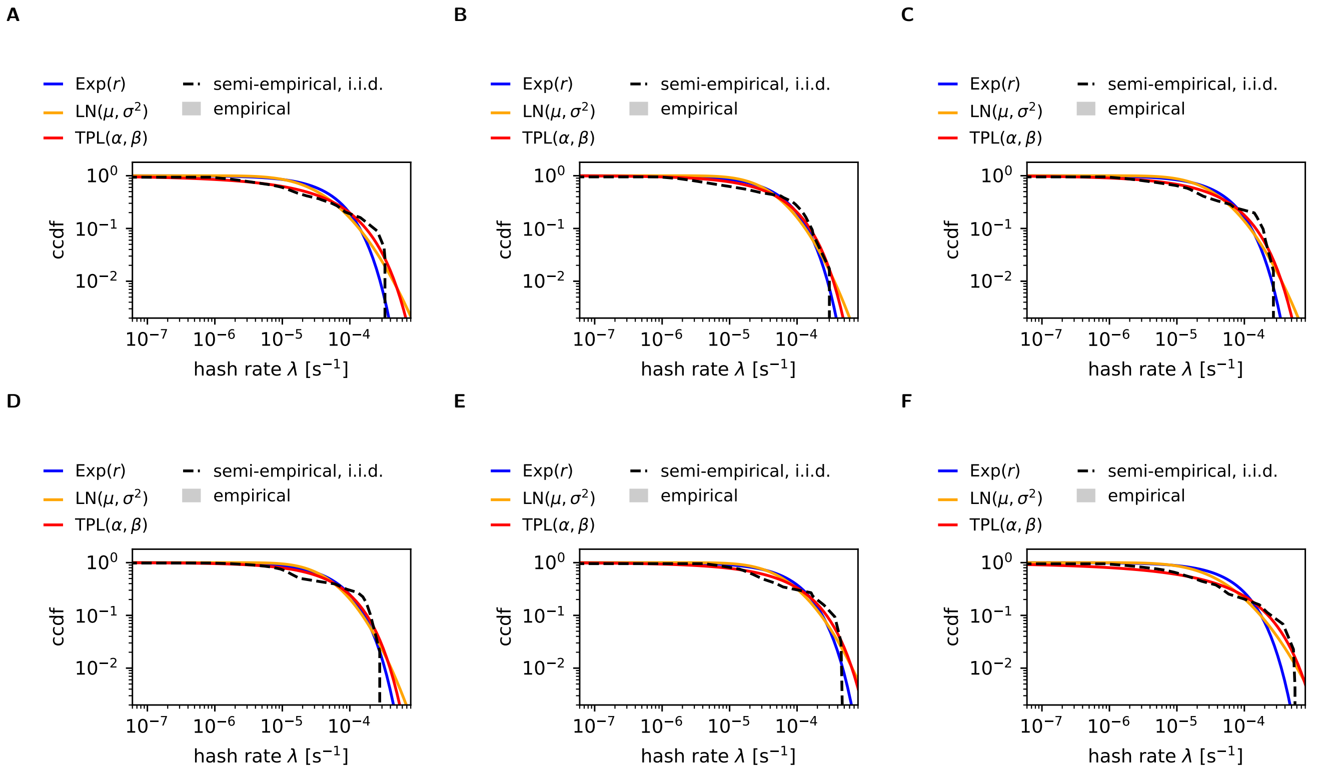

Fig. S5. Complementary cumulative distribution function (ccdf) of hash rates' empirical distribution and null distributions—exponential, log normal, and truncated power law—fitted through the method of moments Eq. 15 in *Research Article* in selected observation periods. **A**: block 364896-366911. **B**: block 465696-467711. **C**: block 566496-568511. **D**: block 667296-669311. **E**: block 768096-770111. **F**: block 868896-870911.

References

1. M Mitzenmacher, A Brief History of Generative Models for Power Law and Lognormal Distributions. *Internet Math.* **1**, 226–251 (2004).
2. Y Gao, C Campajola, N Vallarano, AS Teixeira, CJ Tessone, Heterogeneity- and homophily-induced vulnerability of a P2P network formation model: the IOTA auto-peering protocol. *arXiv preprint arXiv:2401.12633* (2024).
3. I Makarov, A Schoar, Blockchain Analysis of the Bitcoin Market. *SSRN 3942181* (2021).
4. C Campajola, et al., The Evolution Of Centralisation on Cryptocurrency Platforms. *arXiv preprint arXiv:2206.05081* (2022).
5. C Campajola, M D'Errico, CJ Tessone, MicroVelocity: rethinking the Velocity of Money for digital currencies. *arXiv preprint arXiv:2201.13416* (2022).
6. SM Burroughs, SF Tebbens, Upper-truncated power laws in natural systems. *Pure Appl. Geophys.* **158**, 741–757 (2001).
